# Open Access As Public Policy

**DOI:** 10.1371/journal.pbio.0020353

**Published:** 2004-09-21

**Authors:** Andy Gass

## Abstract

Governments around the world have turned their attention to the issue of taxpayer access to primary research literature stemming from publicly funded investigations

The global debate over access to primary research literature heated up this summer, fueled by a slew of congressional and parliamentary recommendations, claims of political victory by critics and proponents of open access, and redoubled lobbying efforts on every side of the issue. After months of often dizzying rhetoric from virtually all camps, one concrete development has indisputably emerged from the fray: governments around the world have begun to take an interest in the question of who can and can't read the results of the scientific research they fund. “We are convinced,” concluded a recent report from the Science and Technology Committee of the United Kingdom's House of Commons, “that the amount of public money invested in scientific research and its outputs is sufficient to merit Government involvement in the publishing process” (House of Commons Science and Technology Committee 2004). United States National Institutes of Health (NIH) Director Elias Zerhouni echoed the British assessment, asserting that “the public needs to have access to what they've paid for,” in a July 28 meeting of stakeholders in scientific and medical publishing. “The status quo,” he added, “just can't stand” (Park 2004).

While such pronouncements may sow fear in the hearts of some scientists and publishers, concerns that governments are poised to tell researchers where or how to publish seem largely unfounded. Both the UK report and rumblings from the US government suggest that any legislative dictates on access to scientific literature are likely to be structured to minimize potentially deleterious implications for established, subscription-based journals, for-profit and not-for-profit alike. Mandates for open access to articles summarizing the results of publicly funded research would not be mandates for scientists to submit work only to the handful of journals, like *PLoS Biology* and *PLoS Medicine,* that currently make their content immediately free online in centralized repositories. A US House of Representatives Committee on Appropriations, for example, recently passed language that would allow many, though not all, publishers six months between the date of publication of NIH-funded research articles and the date of their deposition in a free-to-use archive. (At the time of this writing, the bill is awaiting further discussion in the House and Senate.)

In any case, it is a perfectly reasonable premise that governments should attach conditions to grants mandating public access to resulting peer-reviewed, published articles. Making funding for research contingent on the results of the work being disseminated as widely as possible is hardly a revolutionary proposition. All funders expect, of course, that scientists won't simply stash their findings in a desk drawer. Most, like NIH, include in their mission statements clauses about “fostering the communication of medical and health sciences information” (NIH 2004). The US National Library of Medicine, a division of NIH, goes so far as to provide the infrastructure for hosting and storing the full texts of journal articles online, in the form of PubMed Central. Actually requiring that publicly funded works be *included* in publicly funded electronic archives like PubMed Central, as the US Congress might, would be less a paradigm shift or a radically interventionist mandate than a sensible extension of existing policy for most governments and their funding agencies.

Increasingly, it seems, this is the view being adopted by policy makers—that it is the status quo, rather than prospective policy revision, that is anomalous or hard to justify. “We would be very surprised,” the Science and Technology Committee notes, “if Government did not itself feel the need to account for its investment [in research] in the publishing process. We… hope that this report will be a catalyst for change” (House of Commons Science and Technology Committee 2004).

As a matter of sheer principle, it strikes many people as odd that “anyone can download medical nonsense from the Web for free, but citizens must pay to see the results of carefully conducted biomedical research that was financed by their taxes,” as Rick Weiss noted on the front page of the *Washington Post* last year (Weiss 2003). While neither the US nor the UK has yet to legislate a remedy for this *prima facie* paradoxical state of affairs, both appear ready to address the issue systematically, and—more significantly—with the input of a wide range of affected constituents: scientists, publishers, librarians, patient advocates, text-miners, entrepreneurs, and more. The Science and Technology Committee (2004) report was the product of a seven-month investigation, featuring some 127 submissions of written evidence and four days of oral testimony from the likes of Nature Publishing Group, Reed Elsevier, and indeed, the Public Library of Science. NIH has promised a period of public comment on its plan for implementing the Appropriations Committee's requirement before moving forward, in addition to the information-gathering meeting of publishers in July and subsequent meetings hosted by Dr. Zerhouni. All told, the current spate of government attention to the issue of public access to research results seems methodical, inclusive, and likely to prove productive for scientific communities and the public.
